# 

**Published:** 1869-10

**Authors:** 


### BUSINESS NOTES.

LADIES—ALL OF YOU.—Seeley, the dry goods man, at 36 Opera House, wants to see you. Has just received his fall dress goods—go see them.

MEDICINES CAREFULLY COMPOUNDED at Kellogg & Converse's Drug Store, No 145 Water street.

PLUMBING AND GAS FITTING done neatly and scientifically at E. H. COOK & Co.'s. Orders from out of the city attended to.

ONE DOLLAR—Will buy a beautiful package of French stationery, stamped with your initial, at Hall Brothers.

"TABLE FIXINGS."—Go to Uri Mills & Son, No. 19 Lake street, for your family stores. They keep the nicest tea, coffee, sugar and provisions in the market. Have them supply your table.

---

THE POPULAR STORE in this city, is the one presided over by Barney, Stowell & Davis, at 38 Lake street, Opera House Block. They sell oceans of goods, and are continually receiving new ones.

---

FALL FRUITS.—Grapes, peaches, apples, and other fruits in great abundance, and perfectly fresh, to be had of J. J. Thro, at the "Post Office Grocery." Buy your peaches for canning there.

---

PREPARE FOR WINTER.—Get your houses in order. Place the old carpets where less use will fall upon them, and buy new ones for the family rooms. Durland & Pratt will supply you from their large stock of carpets, at 132 Water street.

---

ENTERPRISING.—Hall Brothers are not only building the handsomest block of stores in the city, but mean to have them crammed full of school books, stationery, wall paper, window shades, and everything of that sort, as they now have their old store. They will now sell their old stock cheap, so as to fill their new house with entirely new goods. Call and see them.

---

FIRE UP!—Prepare for cold weather—go to E. H. Cook & Co.'s and buy your stoves and furnaces. Send to them for globe valves and steam whistles. Order them to do your steam and gas fitting. Buy your plumbers' goods of them, and select your gas fixtures in their establishment. They occupy three large stores—101, 103 and 105 Water street, and have the largest assortment of the articles mentioned, outside of New York city.

---

MEDICINES. — Druggists and physicians, throughout the country, are reminded that the place to buy pure medicines and drugs, is of Wm. H. Gregg & Co., wholesale druggists of this city. Dr. Gregg is an accomplished chemist, manufactures many tinctures, etc., and practices as an analytical chemist. Send to them for a price list of medicines and drugs.

---

PLATED SILVER WARE.—Collingwood & Strang have an unusually large assortment of the best brands of silver plated and solid silver wares on hand. Many of the designs are wonderfully beautiful. They make a specialty of silver goods, and sell the very best, most, and cheapest in the city. Ask Henry to show you some of his beautiful tea sets; they are well worth a visit to look upon.

---

BREECH-LOADING GUNS.—We have in our possession one of Parker's breech-loading shot guns, which, for simplicity of construction, neatness of finish, accuracy of shooting, and the low figure at which it is afforded, surpasses any breech-loader we have ever seen. The fifty dollar gun, manufactured by Parker Brothers at West Meriden, Conn., is as effective as any three hundred dollar breech-loader in the country. We like it—hence our opinion.

---

HANDSOME CUTLERY.—We have discovered where to procure the best and cheapest cutting implements in the market. If you desire a fine razor—one that will cut without pulling; a pair of scissors that trim, without chawing; a carving-knife that will incise, without mangling; a pocket-knife that will whittle, without breaking; in short, if you are in need of any sort of an edge tool, and a reliable one, go to ROWLAND & BEADLE'S, on Water street. They can supply you with anything in that line, and of the very best quality too—we've tried them—and know.

---

INGENIOUS DENTAL OPERATIONS.—We have had occasion, several times, to test the ingenuity of Dr. Brownell, in supplying plates for deformities of the mouth and jaw. He has just completed a plate to cover an opening in the roof of the mouth of a young lady patient of ours, which fits so neatly, and answers the purpose for which it is intended, so nicely, that we cannot forbear making this note of it. Hope the doctor will pardon us for referring to him in this public manner, and also for recommending all in need of first-class dental operations, to call at his rooms, No. 147 Water street.

---

WALL PAPER.—Hall Brothers, at 128 Water street, are receiving their usual stock of fancy wall paper for the fall trade. Everybody desires to have their home look cheerful and pleasant during the long winter evenings, when most of our time is spent within doors; and nothing can contribute more to that end than handsomely decorated walls. It is a matter of health, too, as well—paper becomes nasty by long and continued use, and should be replaced by new in order to render the rooms cleanly and healthful.

Go to the Messrs. Hall and select from the beautiful samples which they will cheerfully show you.

